# Age-dependent patterns of microRNA RISC loading

**DOI:** 10.18632/aging.100692

**Published:** 2014-10-03

**Authors:** Andrey Grigoriev, Nancy M. Bonini

**Affiliations:** ^1^ Department of Biology, Center for Computational and Integrative Biology, Rutgers University, Camden, NJ 08102, USA; ^2^ Department of Biology, University of Pennsylvania, Philadelphia, PA 19104, USA

Two classes of short RNAs have been identified as sequence-specific posttranscriptional regulators of gene expression, acting via RNA-induced silencing complexes (RISCs). They are termed small interfering RNAs (siRNAs) and microRNAs (miRNAs). Despite initial discovery from unrelated studies, these RNA classes are related in their biogenesis and assembly into RISC RNA–protein complexes, and are able to regulate gene transcripts negatively in diverse eukaryotes (Ambros, Nature, 2004, 431:350-355; Bartel, Cell, 2004, 116:281-297; Czech and Hannon, 2011, Nature Rev Genet, 21:19-31).

miRNAs are small RNA molecules of 20–24 nucleotides in length generated from endogenous transcripts and produced by Dicer, a double-stranded RNA-specific enzyme of the RNAse III family. They are typically loaded into the Ago1-RISC complex, where they trigger the silencing of complementary mRNA targets, acting through translational repression and mRNA cleavage. Following the discovery of the first miRNA, lin-4, in the nematode *Canaerobditis elegans*, hundreds of miRNAs have now been identified across multiple species, from plants to humans. miRNAs affect a multitude of biological processes at different stages, from development to aging. Their involvement in aging was initially measured by related changes in abundance. Coincidentally, lin-4 in *C. elegans* was later found to play a critical role, like miR-34 in *Drosophila,* in organismal and brain aging (Boehm and Slack, Science, 2005, 310:1954-1957; Kenyon, Ann NY Acad Sci, 2010, 1204:156-162; Smith-Vikos and Slack, J Cell Sci, 2012, 125: 7-17; Liu et al., Nature, 2012, 482:519-523). siRNAs are also small RNAs of 21–25 nucleotides in length produced by Dicer. siRNAs are loaded into Ago2-RISC and trigger the silencing of their complementary RNA targets, classically functioning in the antiviral response. Small RNAs loaded into Ago1-RISC and Ago2-RISC differ in modification: small RNAs loaded into Ago1-RISC remain unmodified whereas those loaded into Ago2-RISC undergo 2′-*O-*methylation at the 3' terminal ribose (Czech et al., Mol Cell, 2009, 36:445-456; Okamura et al., Mol Cell, 2009, 36: 431-444; Ghildiyal et al., RNA, 2010, 16:43-56). Methylation occurs on nearly all miRNAs and siRNAs in plants and protects the small RNAs from uridylation and degradation (Zhao et al., RNA Biol, 2012, 9: 1218-1223). It also plays a protective role in animals, as loss of 2′-*O-*methylation leads to destabilization, tailing and trimming of siRNAs (Kurth and Mochizuki, RNA, 2009, 15: 675-685; Ameres et al., Science, 2010, 328: 1534-1539; Kamminga et al., EMBO J, 2010, 29: 3688-3700).

Previous studies on the role of *Drosophila* miR-34 in aging and age-associated brain degeneration highlighted an intriguing pattern: miR-34 isoforms of different lengths were seen, with only the short isoform accumulating with age in the brain (Liu et al., Nature, 2012, 482:519-523). These forms are generated by a novel 3′-to-5′ exonuclease, Nibbler, which trims miR-34 as well as a number of other miRNAs, generating a diversity of isoforms differing by length at the 3'end (Liu et al., Curr Biol, 2011, 21: 1888-1893; Han et al., Curr Biol, 2011, 21:1878-1887). The finding of a pattern of miR-34 isoforms with age raised the potential importance of regulation of miRNA length, in addition to miRNA abundance.

We therefore explored the diversity of miRNA length and isoform pattern with age. By examining a number of miRNAs that present with a diversity of isoform lengths, we unexpectedly found a diversity of isoform patterns with age. In particular, a number of miRNAs showed an increase in the levels of the longest isoform with age, rather than the shortest as with miR-34. Detailed analysis revealed that this was likely due to protection of these miRNAs at the 3'end by methylation. Specifically, although most miRNAs are loaded into Ago1-RISC and remain unmodified, a subset are loaded into Ago2-RISC and become 2′-*O-*methylated and protected from Nibbler modification with age. This leads to the change in isoform abundance with age. Indeed, the partitioning of miRNAs into the different RISC complexes—Ago1-RISC and Ago2-RISC—appears to change significantly with age.

Thus our data indicates there is a complex and unexpected diversity of isoform patterns in young (3d) compared to older (30d) flies. The age-associated increase of some *Drosophila* miRNAs reflects an increase in 2′-*O-*methylation of select isoforms; associated with this phenomenon is increased loading of select miRNAs into Ago2, but not Ago1, with age. The animal is thus regulating the partitioning of miRNAs between the RISC complexes with age. To address the biological significance of this shift in loading of miRNAs, *Hen1* and *Ago2* mutants that lack 2'-O-methylation were examined; these animals showed brain degeneration and reduced lifespan.

Key questions include how and why miRNA partitioning between the RISC complexes changes with age for select miRNAs. Does the “how” involve interactions of specific factors with Ago1 and/or Ago2 that alter with age? Does the “why” involve the aging organism actively adjusting the efficiency of target gene expression by shifting miRNAs towards Ago2-RISC and away from Ago1-RISC, for ongoing or upcoming age-associated stresses? Or could the accumulation of miRNAs in the Ago2-RISC be a by-product of increased stability of their 2′-*O-*methylated isoforms? This phenomenon can also be viewed in the context of an age-associated increase of transposon expression, neuronal decline and shorter lifespan noted in *Ago2* mutants (Li et al., Nat. Neurosci, 2013, 16:529-531; Perrat et al., Science, 2013, 240:91-95). Does the accumulation of miRNAs in Ago2-RISC impact the efficiency of these and other biological phenomena that depend on Ago2-RISC, for example, the action of siRNAs and endo-siRNAs in the aging organism?

